# Care trajectories of surgically treated patients with a prolactinoma: why did they opt for surgery?

**DOI:** 10.1007/s11102-023-01346-z

**Published:** 2023-09-10

**Authors:** Victoria R. van Trigt, Ingrid M. Zandbergen, Iris C. M. Pelsma, Leontine E. H. Bakker, Marco J. T. Verstegen, Wouter R. van Furth, Nienke R. Biermasz

**Affiliations:** 1 Division of Endocrinology, Department of Medicine, Center for Endocrine Tumors Leiden, Leiden University Medical Center, Leiden, The Netherlands; 2 Department of Neurosurgery, Leiden University Medical Center, University Neurosurgical Center Holland, Leiden, The Netherlands

**Keywords:** Prolactinoma, Transsphenoidal surgery, Care trajectory, Multidisciplinary counselling, Treatment indications

## Abstract

**Purpose:**

To describe care trajectories in patients with prolactinoma, aiming to clarify the rationale for surgery.

**Methods:**

Retrospective observational cohort study of consecutive patients with prolactinoma undergoing surgery from 2017 to 2019 at the referral center (RC), prior to surgery being considered a viable treatment option (i.e. PRolaCT study). Demographics and clinical data (type and duration of pretreatment and surgical indications, goals, and outcomes) were collected from patient records. Care trajectories were divided into three phases: (1) diagnosis and initial treatment, (2) endocrine treatment at the RC, and (3) surgical treatment.

**Results:**

40 patients were included (31 females (77.5%), median age 26.5 (14–63) years. Indications for surgery were dopamine agonist (DA) *intolerance* (n = 31, 77.5%), *resistance* (n = 6, 15.0%), and *patient/physician preference* (n = 3, 7.5%). Patients were pretreated with DA (n = 39 (97.5%)), and surgery (n = 3 (7.5%)). Median disease duration at surgery was 4 (0–27) years. Primary surgical goal was total resection in 38 patients (95.0%), of which biochemical remission was achieved 6 months postoperatively in 23 patients (62.2%), and clinical remission in 6 patients (16.2%), missing data n = 1.

**Conclusion:**

Care trajectories were highly individualized based on patient and tumor characteristics, as well as the multidisciplinary team’s assessment (need for alternative treatment, surgical chances and risks). Most patients were pretreated pharmacologically and had broad variation in timing of referral, undergoing surgery as last-resort treatment predominantly due to DA intolerance. High quality imaging and multidisciplinary consultations with experienced neurosurgeons and endocrinologists enabling treatment tailored to patients’ needs were prerequisites for adequate counseling in treatment of patients with prolactinoma.

**Supplementary Information:**

The online version contains supplementary material available at 10.1007/s11102-023-01346-z.

## Introduction

Prolactinomas are the most common hormone-secreting pituitary adenomas. Pharmacological treatment with dopamine agonists (DAs) is first line treatment. DA treatment is effective in about 83% of patients, however, > 26% reported side effects, e.g. gastrointestinal complaints, postural hypotension, mood-related effects, and, more rarely, impulse control disorders [1, 2]. DA side effects—long overlooked and underreported—may affect health related quality of life (HR-QoL) in a subgroup of patients. Individual stories, as described in Outline 1, made our multidisciplinary team (MDT) critically reappraise prolactinoma management. Surgery is an alternative treatment, which was generally only considered in case of mass-effects, DA intolerance, or resistance [3], although this paradigm has shifted [1, 4].

**Outline 1:**
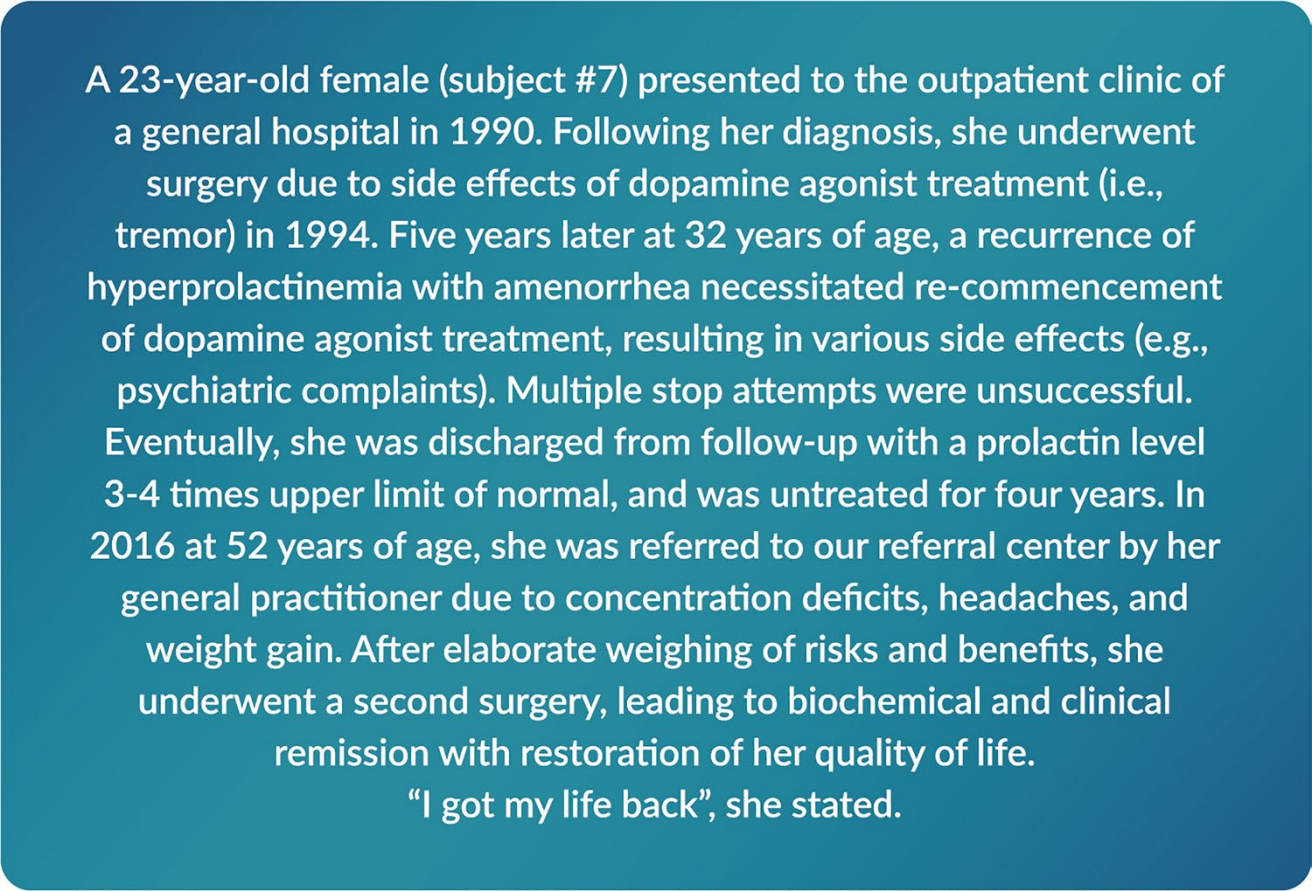
Case vignette of a female patient who underwent surgery for her prolactinoma remnant at the referral center

At our referral center (RC), a dedicated multidisciplinary care pathway for patients with pituitary tumors has been developed, according to Value-Based Health Care (VBHC) principles [5]. Structured outcome evaluation, multidisciplinary counseling, and shared decision-making—involving adequate appreciation of patient- and adenoma-specific characteristics—are important aspects of this care pathway. These processes require a holistic view on patient care and extensive experience with all treatment modalities, including periodical outcome evaluations. The benefits and risks, or adverse effects of pharmacological treatment versus the medical need, chance of total resection (TR), and the risk of surgical complications are weighed carefully for each individual case [6].

Recent literature has suggested that surgery may be a viable cost-effective first-line therapy for prolactinomas of limited size [1, 7, 8], with fast recovery of disease burden and HR-QoL [5]. The increased interest in surgery has resulted in ongoing international debates, rising numbers of referrals to the RC for surgical counseling, and initiation of prospective comparative studies (including PRolaCT [9, 10]). Generally, patients opting for surgery have undergone long-term DA treatment, possibly complicating surgery due to prolactinoma shrinkage and induration, or have specific, biased positive or negative ideas about surgical possibilities, complications, and outcomes.

To gain insight and understanding in factors influencing referral patterns and treatment decisions, care trajectories and clinical treatment considerations were systematically analyzed. With these observations, this study aimed to clarify the rationale behind surgical treatment in a consecutive surgical cohort of patients with prolactinoma in 2017–2019, prior to the paradigm shift from DA treatment being cornerstone to surgery being a potential first-line treatment option.

## Methods

### Participants and study design

This retrospective observational cohort study based on a chart review describes the care trajectory, from diagnosis to surgery, of 40 consecutive patients who underwent surgery for a prolactinoma at the LUMC (referred to as *the RC*) between January 1, 2017 and June 1, 2019 without being included in the PRolaCT-study (NCT:04107480) [9]. Patients for whom surgery was not elective (i.e. cerebrospinal fluid leakage (n = 1), progressive visual field defects (n = 2) and acromegaly (n = 1)) were excluded. Data were collected prospectively as part of standard care in the VBHC care pathway, with additional data regarding treatment decision and referral details being retrospectively retrieved from the electronical patient records (EPR). The need for informed consent for the standard and additional data collection was waived by the Scientific Committee (research protocol G19.011).

### Data collection

Two authors (VRvT and IMZ) analyzed all patient records separately and subsequently composed one combined care trajectory, describing clinical, radiological, and biochemical parameters from diagnosis until either the moment patients were lost to follow-up, or the February 10, 2023. Disagreements were resolved by discussion, and in case of uncertainty or persistent disagreement, the majority vote was selected through consultation of a third author (ICMP) to reach consensus. The care trajectories were reviewed by a fourth author and member of the treating MDT (NRB) for accuracy. The care trajectory was divided in three phases, with different subphases, as described prior [11, 12]: (1) before referral to the RC: first hospital presentation, treatment before referral, (2) endocrine treatment at the RC: first presentation to the RC, pharmacological treatment, decision-making regarding surgical tumor removal, and (3) surgical treatment at the RC: patient characteristics at time of surgery, surgical removal and outcomes, and long term follow-up and postoperative treatment.

### General care pathway

All patients were treated at the outpatient clinic of the RC (an RC for pituitary care and a nationally and internationally endorsed center of expertise within the European Reference Network on Rare Endocrine Conditions (Endo-ERN) [13]) following a predefined VBHC care pathway, complying to international guidelines, as described prior [3, 5]. Furthermore, data regarding the trajectories before referral to the RC were collected from referral letters and notes in the EPR. In the Dutch healthcare system, the general practitioner (GP) provides primary care, and is generally the first healthcare professional patients contact with health-related problems. The GP assesses patients' symptoms and refers to a regional hospital (RH) if necessary (secondary care). Only in cases of emergency, a patient is referred to a hospital directly without a GP’s assessment. RCs (tertiary care) provide highly specialized care and may be accessed through referral by a GP or physician from an RH.

Generally, patients were started on standard therapy (DAs) after initial evaluation, (if not already receiving treatment) or switched to a different DA in an attempt to eliminate side effects. Additionally, the pituitary axes were evaluated, and any deficiencies were treated. In males with microadenomas, substitution of the gonadotropic axis instead of DA therapy was considered in case of DA side effects. Surgery was considered in case of persistently impaired HR-QoL, contra-indications for DA or strong patient/physician preference. In selected cases with indeterminate MRI results, functional imaging by MET-PET/MRI was performed to more clearly visualize the localization or extension of the lesion, as described prior [14]. All patients considered for surgery were discussed during weekly multidisciplinary meetings preoperatively, immediately postoperatively, two weeks, and six months postoperatively. During the preoperative meetings, the need for (surgical) treatment, estimated chance of achievement of the (surgical) goals and risks were systematically discussed, documented (in the EPR and a database), and reevaluated postoperatively. Pre- and postoperative evaluation included dynamic testing of pituitary axes on clinical indication. All patients in our cohort underwent surgery by two neurosurgeons from a team of three dedicated, experienced pituitary neurosurgeons [6].

### Study parameters

#### Patients and disease characteristics

The following demographic and clinical parameters were collected from the EPR: sex, age, disease duration, serum prolactin level, and pituitary hormonal deficiencies. Hormonal deficiencies were defined and treated according to available guidelines, as described prior [15–17]. The following tumor characteristics were recorded based on the reports of diagnostic and preoperative MRI scans: tumor size, cavernous sinus invasion (CSI, KNOSP > 2), and chiasmal compression.

#### Treatment prior to referral to the RC

The following information on prolactinoma care prior to referral was retrieved from referral letters: time of first prolactinoma-related consultation, department of first presentation, symptoms at first presentation, and type, duration, and side effects of previous treatment(s). Treatment duration was categorized as < 6 months, 6 months-2 year, 2–10 years, and > 10 years. DA side effects were defined as symptoms known as DA side effects (e.g. gastro-intestinal complaints, mood disturbances and headaches), not caused by hyperprolactinemia, prolactinoma mass effects, or known comorbidities. Symptoms at first presentation were: menstrual cycle disturbances, galactorrhea, headache, psychological complaints, reduced libido, weight gain, subfertility, gynecomastia or delayed puberty. Reasons for RC referral were deduced from referral letters and reports of the first consultation at the RC, being: expertise, uncertain diagnosis, pregnancy wish, dissatisfaction with care, and preference for surgery.

#### Treatment at the RC

The following parameters were calculated based on the EPR: time between diagnosis and first presentation to the RC, number of preoperative consultations with a neurosurgeon before surgery at the RC, time between first consultation at the RC and definitive decision for surgery, duration and type of treatment, and postoperative follow-up duration.

Based on collected data, three indications for surgery were defined: (1) DA intolerance, (2) DA resistance, (3) preference for surgery. Patients were considered *DA intolerant* if side effects were unacceptable in the opinion of both the patient and treating specialist. *DA resistance* was defined as persisting hyperprolactinemia and/or no tumor shrinkage whilst on the maximum tolerated DA dose (being ≥ 2 mg/week of cabergoline, ≥ 7.5 mg/day of bromocriptine [18] or ≥ 150mcg/day of quinagolide). Patients were assigned to the *preference* category if there was no DA intolerance or resistance, and if the patient’s and/or physician’s preference for surgery was explicitly noted as indication in the EPR.

The primary goal (restoration of hormonal excess or reduction of DA dose) and surgical technical goals (debulking or TR) were deduced from the EPR. The chance of achieving these goals was categorized as optimal, if a clear adenoma could be visualized on MRI without extension into surrounding structures or known fibrosis (based on prior surgical findings), or suboptimal if not complying to these criteria. Biochemical remission was defined as normalization of prolactin (< 1.0xULN). Clinical remission was defined as restoration of gonadal function and resolution of complaints (i.e. no additional treatment needed) without normalization of prolactin. Patients who were not in biochemical or clinical remission were perceived to have persistent disease. Postoperative complications were assessed 6 months postoperative, as complications persisting ≥ 6 months postoperative were considered permanent.

### Hormonal assays

Prolactin levels were measured on a Cobas E602 immuno-analyzer using the Elecsys Prolactin II kit of Roche Diagnostics, Mannheim Germany. Measurement range was 0.047–470 ng/mL (1.00–10000 mIU/L). No high dose hook effect was found up to 12690 ng/mL (270000 mIU/L). At 49.7 ng/mL the variation coefficient (VC) was 2.55%, and at 5.9 ng/mL VC was 2.38%. Both values were based on 400 + measurements of internal quality control samples.

### Data description

IBM SPSS statistics 25 (IBM Corp. Armonk, NY, USA) was used for data descriptions. Data was reported as median (range) for continuous variables, and frequency (percentage) for dichotomous variables. Solely the absolute values are presented in the Manuscript. The percentages are presented in the (Supplementary) Tables.

## Results

### Clinical characteristics and surgical indications

#### Clinical characteristics at diagnosis

Forty patients, of whom 31 females, with median age of 26.5 (14–63) years, were included. Patient characteristics at diagnosis are shown in Table 1. The diagnostic MRI (performed/available for 39/40 patients), showed a microadenoma in the majority of patients (24/39), whereas 13/39 patients had a macroadenoma and 2/39 patients had no visible adenoma. A detailed chronological overview of the trajectory per patient is presented in Table 2. When a specific patient is discussed, the patient is referred to by the patient ID (‘#X,’ as presented in Table 2). Two male patients had certain CSI KNOSP > 2 (#17, #32), of whom one patient also showed certain chiasm compression (#17). Possible chiasm compression was present in patient #29.

**Table 1:** Demographic data and MRI results at diagnosis

	All patients	Females	Males
	N = 40	N = 31 (77.5%)	N = 9 (22.5%)
Age (years)	26.5 (14–63)	25 (14–41)	31 (18–63)
MRI at diagnosis	N = 39^a^	N = 30 (76.9%)^a^	N = 9 (23.1%)
Tumor size			
No adenoma visible	2 (5.1%)	2 (6.7%)	0 (0.0%)
Microadenoma	24 (61.5%)	19 (63.3%)	5 (55.6%)
Macroadenoma	13 (33.3%)	9 (30.0%)	4 (44.4%)
Giant adenoma	0 (0.0%)	0 (0.0%)	0 (0.0%)
CSI (> KNOSP 2)			
Certain	2 (5.1%)	0 (0.0%)	2 (22.2%)
Unknown	3 (7.7%)	3 (10.0%)	0 (0.0%)
Optic chiasma compression			
Certain	1 (2.6%)	0 (0.0%)	1 (11.1%)
Possible	1 (2.6%)	0 (0.0%)	1 (11.1%)
Apoplexy			
Certain	2 (5.1%)	2 (6.7%)	0 (0.0%)
Possible	1 (2.6%)	1 (3.3%)	0 (0.0%)

Demographical data and MRI results at diagnosis for all patients and for females and males separately. Values are presented as median (range) or number (percentage)

*CSI* cavernous sinus invasion, *DA* dopamine agonist

^a^The MRI scan at diagnosis was unavailable in one patient who was diagnosed abroad

**Table 2:**
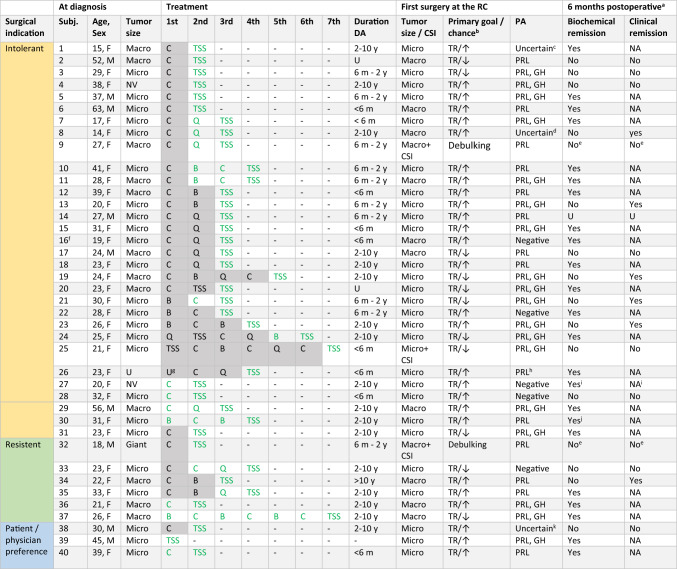
Demographics and treatment details per patient

Overview of demographical data, tumor characteristics, treatment details and outcomes per patient. The treatment trajectory till the first surgery at the RC was depicted. Patients may have undergone additional treatment after the first surgery at the RC. Compression of the optic chiasm was present in none of the patients at time of surgery. No permanent complications occurred. Biochemical remission was defined as normalization of prolactin. Clinical remission was defined as restoration of gonadal axis and resolution of symptoms, i.e. no indication for further treatment. *B* bromocriptine, *C* cabergoline, *CSI* cavernous sinus invasion, *F* female, *GH* growth hormone, *M* male, *Macro* macroadenoma, *Micro* microadenoma, *NV* not visible, *PA* histopathology, *PRL* prolactin, *Q* quinagolide, *RC* referral center, *subj*. subject, *TR* total resection, *TSS* transsphenoidal surgery, *U* unknown, *↑* optimal surgical chance of total resection, *↓* suboptimal surgical chance of total resection, 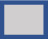 Treatment undergone before referral to the RC, 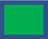 Treatment undergone at the RC

^a^6 months after the first surgery performed at the referral center

^b^Chance of achieving total resection for the patients in whom total resection was the primary surgical goal

^c^Hemorrhage and tissue that could be preexisting pituitary or adenoma with positive staining for ACTH, growth hormone and to a lesser extent prolactin

^d^No certain adenoma, small area with increased expression of prolactin and growth hormone

^e^Remission not expected as the goal of surgery was debulking

^f^Patient is a BAP1 gene mutation carrier

^g^Medication started in Poland, unknown which agent bold Treatment undergone at the RC

^h^Dubious expression of growth hormone italic Treatment undergone before referral to the RC

^i^Remission status was measured 11 months postoperative

^j^Remission status measured 2 months postoperative, as the patient was lost to follow-up from this point on

^k^Uncertain adenoma, possible apoplexy

#### The care trajectory

The heterogeneous care trajectories prior to surgery are summarized below based on the predefined phases of the care trajectory.

### Phase 1: the care trajectory: before referral to the RC

#### First hospital presentation

**Fig. 1:**
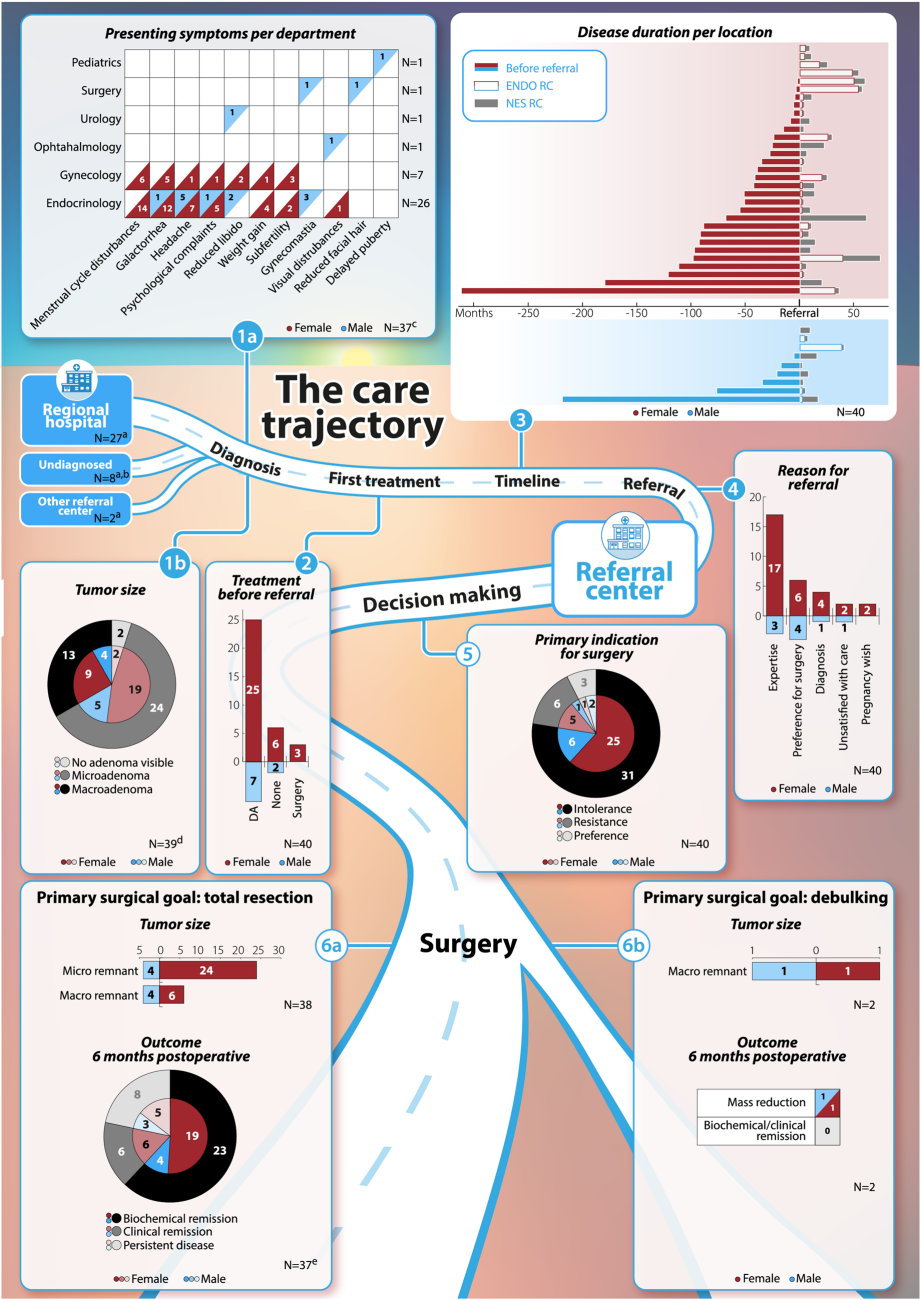
Summary of care trajectory for all patients. A summary of demographics, radiological data and clinical outcomes at different timepoints (1–6) throughout the care trajectory are reported for the entire cohort, as well as for females and males separately. Before referral to the referral center: *Timepoint 1a *Diagnosis: presenting symptoms per department of first hospital presentation. *Timepoint 1b* diagnosis: tumor size at first presentation. *Timepoint 2* First treatment: treatment undergone before referral to the referral center. *Timepoint 3* Timeline: duration of treatment per location. *Timepoint 4* at time of referral: primary indication for referral. At the referral center: *Timepoint 5* decision making: primary indication for sugery. *Timepoint 6a* information concerning patients who underwent surgery aiming for total resection: tumor size at time of surgery and surgical outcomes 6 months postoperative. *Timepoint 6b *information concerning patients who underwent surgery aiming for debulking: tumor size at time of surgery and surgical outcomes 6 months postoperative. *DA* dopamine agonist, *ENDO RC* Department of Endocrinology at the referral center, *macro* macroadenoma, *micro* microadenoma, *N* number of patients, *NES RC* Department of Neurosurgery at the referral center, *RC* referral center. (a) Data missing for 3 females, (b) 8 patients were undiagnosed before referral to the referral center, therefore diagnosis and the first treatment took place at the referral center. (c) Data missing for 1 female. (d) Data missing for 1 male

Twenty-seven patients initially presented to an RH, whereas 10 patients presented directly to an RC (*the RC* n = 8, other n = 2). For three females (#25, #26, #30), details on the type of hospital of presentation were unavailable due to presentation outside the Netherlands. As shown in Fig. 1, female patients presented at either the Departments of Endocrinology (n = 21), or Gynecology (n = 7), with the most common presenting symptoms being menstrual cycle disturbances and galactorrhea. Male patients presented at a wider variety of departments: Department of Endocrinology (n = 5), and Ophthalmology, Urology, Surgery, or Pediatrics (n = 1 each). Initially, prolactinoma diagnosis was unclear in three patients: patient #3 was diagnosed with a burnout first (diagnostic delay approx. 3 years), patient #39 underwent surgical removal of gynecomastia (diagnostic delay approx. 4 years), and patient #12 was diagnosed with PCOS (diagnostic delay approx. 3 years).

#### Treatment before referral to the RC

Thirty-two patients were treated with DA prior to referral to the RC. Three patients had additionally undergone surgery, of whom two due to DA side effects (hallucinations and tremor in patient #20 and #24, respectively), and patient #25 because of an apoplexy in a previously undiagnosed prolactinoma, which was followed by DA treatment.

Of DA treated patients, 26/31 patients started with cabergoline. 17/31 patients used only 1 DA, 12/31 patients used 2 DAs, and 2/31 patients were treated with all 3 agents. Details about the first agent(s) were unavailable for patient #26, as treatment commenced outside of the Netherlands. Side effects were reported by 23/30 patients, causing swift discontinuation of the agent in 11/30 patients. #32 showed immediate DA resistance (missing data n = 2).

Patients not treated before referral to the RC had either not yet been diagnosed (5/8), were diagnosed at a Department of Gynecology and referred for treatment (2/8), or objected to DA treatment due aversion to medication (1/8).

### Phase 2: the care trajectory: endocrine treatment at the RC

#### First presentation to the RC

Median time from diagnosis to RC referral was 2 (0–26) years, as shown in Supplementary Table 1. 20/40 patients were referred for expertise, 10/40 patients because of preference for surgery, 5/40 for diagnosis, 3/40 patients due to dissatisfaction with care and 2/40 patients for a pregnancy wish.

#### Pharmacological treatment at the RC

Sixteen patients received DA treatment at the Endocrinology outpatient clinic before referral to the Department of Neurosurgery for multidisciplinary counseling, of which 7/16 patients were treatment-naive. Twenty-four patients were referred directly to the Neurosurgery Department for multidisciplinary counseling upon arrival in the RC. Suppletion of pituitary axes (thyrotropic, corticotropic and gonadal) was initiated or optimized in 3/40 patients at the Department of Endocrinology or Neurosurgery.

In total, all but patient #39 had received pretreatment (at the RC/prior to referral) before undergoing surgery: 14/38 patients used 1 DA, 20/38 patients 2 DAs, and 4/38 patients used all 3 agents (missing data n = 1). 9/37 patients had been treated < 6 months, 10/37 patients for 6 months-2 years, 17/37 patients for 2–10 years, and patient #34 had been treated > 10 years (missing data n = 2). Side effects were reported in 36/39 patients, with headaches being the most common (n = 16), followed by mood disturbances (n = 12), and gastro-intestinal complaints (n = 11). Hallucinations and impulse control disorders were present in 3 (#5, #20, #23) and 1 (#17) patient(s), respectively.

#### Decision-making regarding surgical tumor removal

The need for surgery was high in all patients, as discussed during the preoperative MDT meetings. As shown in Supplementary Table 2, the primary indication for surgery was DA intolerance in 31/40 patients, DA resistance in 6/40 patients, and patient/physician preference in 3/40 patients. Patient #32 was perceived to be resistant, due to persistent invasive growth and unresponsive prolactin levels (> 80.0xULN) despite cabergoline dose increases to 1.75 mg/week, necessitating swift intervention. Notably, 1 intolerant patient (#4) had an additional suspicion of GH co-secretion (IGF-1 + 3.4SD, paradoxical response to oral glucose tolerance test, albeit without acromegaly), and patient #39 with a strong preference for surgery had an additional psychological comorbidity, which was considered a relative contraindication for DA treatment. The main surgical goal was TR in 38/40 patients aiming for prolactin normalization, and debulking with DA dose reduction in 2/40 patients in whom TR was deemed impossible due to CSI (KNOSP 3 and 4 in patient #9 and #32, respectively). The chance of TR was deemed optimal in 26/38 patients*,* and suboptimal in 12/38 patients*,* of whom 4/12 patients (#2, #3, #20, #21) underwent MET-PET/MRI prior to surgery.

The majority of patients with an optimal chance of TR (15/26) had 1 preoperative neurosurgical consultation at the RC (range 1–5), whereas the majority of patients with a suboptimal chance (5/12) had 3 neurosurgical consultations (range 1–4). Median time between the first consultation at the RC and the definitive choice for surgery was 186 (23–2162) days.

### Phase 3: the care trajectory: surgical treatment at the RC

#### Patient characteristics at time of surgery

Median disease duration at time of surgery was 4 (0–27) years. Median preoperative prolactin levels were 4.5 (0.2–81.4) xULN. 9/40 patients used DA (among which all patients with prolactin < 1.0xULN). Comparing diagnostic to preoperative MRI scans, stable tumor volume was observed in 30/39 patients, shrinkage in 4/39 patients, and growth in 5/39 patients. CSI was still present in #32, and new CSI occurred in #25 and #9. None of the tumors compressed the optic chiasm.

#### Surgical removal and outcomes

Data was available for 37/38 patients (#14 missing). Six months postoperatively, 23/37 patients (females n = 19 (63.3%); males n = 4 (57.1%)) in whom TR was intended achieved biochemical remission. Additionally clinical remission was achieved in 6/37 patients (female N = 6, 20.0%) Fig. 1). 8/37 patients had persistent disease (prolactin more than halved n = 5, no improvement n = 3). Remission rate was measured 2 months postoperative in patient #30 who was lost to follow-up from this point on. Mass reduction was achieved in 2/2 patients undergoing debulking, enabling DA dose reduction. Histopathology was confirmative in 32/40 patients, uncertain in 4/40 patients, and negative in 4/40 patients, as shown in Supplementary Table 3. No permanent complications occurred.

#### Long-term follow-up and postoperative treatment

Of the patients with persistent disease at 6 months postoperative, 7/8 patients underwent additional treatment (DA only n = 2, 1–2 reoperation(s) only n = 2, 1–2 reoperation(s) and DA n = 3). Both patients who underwent debulking received additional treatment: DA n = 1, DA and reoperation n = 1. During long-term follow-up, 2/23 patients who were initially in biochemical remission experienced a recurrence, of whom #6 was treated with DAs only and #1 with DAs and reoperation. Of patients initially in clinical remission, 2 (#8, #34) eventually required DA treatment due to recurrence of symptoms and #13 underwent a reoperation.

At last follow-up (median duration 43 (2–71) months), 26/38 patients in whom TR was intended were in biochemical remission, 4/38 in clinical remission, 4/38 had persistent disease and 1/38 recurrence after initial remission. 3/38 were biochemically controlled on DAs. Both patients who underwent debulking had persistent disease despite using DAs.

## Discussion

This study reporting on care trajectories of surgically treated patients with prolactinomas sheds light on clinical considerations leading up to surgery. Despite considerable heterogeneity between individual care trajectories, most patients underwent surgery due to DA side effects after long-term DA treatment. Following referral to the RC, care trajectories varied based on patient preferences, degree of DA intolerance, and tumor and disease characteristics. The MDT’s assessment of surgical possibilities, risks, and estimated chances of achieving the surgical goal influenced counselling and thereby the choice to proceed to surgery. Patients with suboptimal surgical chances and a high need for non-medical treatment due to invalidating DA side effects, or resistance, underwent additional imaging for diagnostic optimization, and thorough counseling to balance advantages and disadvantages, illustrated by a higher number of preoperative consultations. The Endocrinology outpatient clinic served as a potential, albeit not obligatory, stop in the trajectory for optimization of endocrine therapy prior to surgical counseling at the combined endo-neurosurgical clinic.

Our study spans the era during which DAs were considered the cornerstone of prolactinoma treatment, and surgery was not considered standard [3]. The present surgical cohort is therefore a selected group of patients undergoing surgery for (relative) *DA intolerance* (in 75%), *DA resistance*, or *patient/physician preference*. Despite DA intolerance, many patients underwent long-term treatment with (several) DAs before surgical counseling, indicating that surgery was indeed considered last-resort therapy in this period of time. About 25% of patients were treated < 6 months and had a shorter care trajectory before referral to a neurosurgeon, which could be due to either more severe intolerance, or a shifting treatment paradigm with surgery being considered at an earlier stage. In addition to *DA intolerance* and *resistance*, *patient/physician preference* has recently been identified as a valid indication for elective surgery [19–23], as reflected by the present findings. Moreover, early, or first-line surgery has recently been supported by various retrospective observational or cost-efficiency studies [1, 7, 8]. To date, prospective and comparative trials are lacking, as we await the results of ongoing studies [9, 10]. To understand why a subgroup of patients with prolactinoma undergo surgery, knowledge of clinical considerations determining the shared decision-making process concerning prolactinoma treatment is essential.

In the included patients, surgery was preceded by extensive evaluation of the medical need for surgery and shared decision-making by endocrinologists, neurosurgeons, and patients. In absence of comparative trials, multidisciplinary counseling is of utmost importance—a task for dedicated experts at an RC. In our cohort, considerations determining the decision-making process were highly patient-specific, with the time between referral to the RC and the decision of surgical intervention, therefore, varying greatly. During the decision-making process, the need for surgery was mostly determined by symptoms, DA side effects, (relative) contra-indications (e.g. severe depression, suicidality, and impulse control disorders), and resistance to DA treatment. Notably, perception of the severity of side effects is highly subjective, and cannot be objectified. Next, the relationship between side effects, prolactinoma symptomatology, pituitary deficits, and unrelated comorbidity is overly complex, with the origin of the symptoms not always being evident. Thus, before proceeding to surgery, patient and MDT should have a clear shared vision of the need for surgery, and expectations should be managed to fit the expected outcomes of surgery. In case of uncertainty regarding the medical need and expectations, additional diagnostics may be indicated for clarification (e.g. DA withdrawal/restart attempt, dynamic testing hormonal axes, functional imaging).

In addition to scrutinizing the need for surgical intervention, the MDT needs to estimate a patient’s chance of achieving the primary goal (e.g. alleviation of symptoms), surgical technical goal (e.g. TR), and complication risks. During MDT consultations, these chances are weighed with the medical need. In patients with unfavorable risks of complications and chances of achieving the primary goal, surgery should only be considered when the need for alternative treatment is high, as illustrated by our cohort. Based on the present patient cohort, two key prerequisites could be determined for optimal prediction of chances and risks. First, high quality imaging at diagnosis and at surgical counseling (to assess changes over time) was vital to determine the original and current relation with the surrounding structures, and cystic components. Secondly, multidisciplinary outpatient consultation(s) with experienced neurosurgeons and endocrinologists enabled synergistic, in-depth counseling regarding benefits and risks of the treatment options.

Surgical counseling can theoretically take place at multiple treatment phases within the care trajectory: after a short period, i.e. 2–6 months, of DA treatment (for evaluation of DA treatment efficacy and tolerance, including comparison of the chance of remission on DAs to the surgical chance), after an unsuccessful DA withdrawal attempt following 2 years of DAs, according to current guidelines [3] (prolactinomas requiring long-term DA treatment, with milder side effects becoming a burden), or treatment-naïve patients, as proposed in the PRolaCT trial [9]. Despite side effects being reported by 72% of patients in our cohort, the majority received long-term DA treatment before referral to the RC. In light of the changing treatment landscape, the question arises whether earlier/upfront counselling is preferrable, as DA treatment might result in shrinkage and fibrosis, which might be detrimental for surgical success [6, 24, 25]. Nevertheless, the potency of DA treatment should be taken into account during the surgical decision-making process. Optimal timing, therefore, remains a subject for future research.

In our cohort, flexibility and willingness of the treating endocrinologists and neurosurgeons to think beyond the boundaries of current guidelines enabled adapting treatment to the patients’ needs, illustrated by their personal stories. The importance of the patients’ perspective on healthcare in assessment of quality of care has been well acknowledged [11, 12, 26, 27]. EURORDIS and the European Reference Network for patients with a rare genetic tumor risk syndrome (ERN GENTURIS) developed patient journeys as a method for patients to share their experiences and connect clinical guidelines to patients’ needs. Future studies reporting on the patient journeys of patients with a prolactinoma could aid healthcare providers in delivering adequate support, particularly for difficult-to-quantify side effects.

Multiple limitations of this study should be considered. Firstly, information bias may have occurred, due to the retrospective study design. Notably, pituitary axes were only tested dynamically in cases in which clinical suspicion of dysfunction was present. Therefore subclinical deterioration of pituitary function may have been missed. Furthermore, chart reviews were limited to the RC’s EPR. Therefore, details on treatment before referral were not available for all patients. Especially detailed information regarding DA withdrawal attempts and restarts were not clearly described. Because reporting outcomes was not the focus of this study, we used clinical interpretation in addition to strict biochemical outcomes to determine remission. An unmet need is the definition of success for prolactinoma surgery, e.g. how to classify the case with slightly elevated prolactin, but restoration of gonadal function, resulting in spontaneous pregnancy, and without remnant on MRI.

Summarizing, care trajectories of surgically treated prolactinoma patients were highly individualized based on patient and tumor characteristics, as well as the treating specialists' assessment (i.e. the need for alternative treatment, and surgical risks and chances of achieving the surgical goal). In the present cohort of patients with prolactinomas, most patients were exposed to (potentially unnecessarily) long-term DA treatment despite intolerance or resistance. During the inclusion period, the MDT gained experience with counseling and surgery in patients with prolactinomas, leading to the conclusion that high-quality initial imaging and repeated expert multidisciplinary consultations were the most important aspects for adequate treatment counseling. Future studies should assess the patients’ perspective, as well as the optimal timing of surgery, and which patients benefit from surgical intervention.

### Supplementary Information

Below is the link to the electronic supplementary material.

Supplementary file1 (PDF 264 kb)
