# Correction: Care trajectories of surgically treated patients with a prolactinoma: why did they opt for surgery?

**DOI:** 10.1007/s11102-023-01358-9

**Published:** 2023-10-28

**Authors:** Victoria R. van Trigt, Ingrid M. Zandbergen, Iris C. M. Pelsma, Leontine E. H. Bakker, Marco J. T. Verstegen, Wouter R. van Furth, Nienke R. Biermasz

**Affiliations:** 1 Division of Endocrinology, Department of Medicine, Center for Endocrine Tumors Leiden, Leiden University Medical Center, Leiden, The Netherlands; 2 Department of Neurosurgery, Leiden University Medical Center, University Neurosurgical Center Holland, Leiden, The Netherlands

**Correction to: Pituitary (2023) 26:611–621** 10.1007/s11102-023-01346-z

In this article the caption to Fig. 1 was inadvertently omitted. Also, the legend of Table 2 was incomplete. The correct Fig. 1 and Table 2 are given below. The original article has been corrected.

**Fig. 1:**
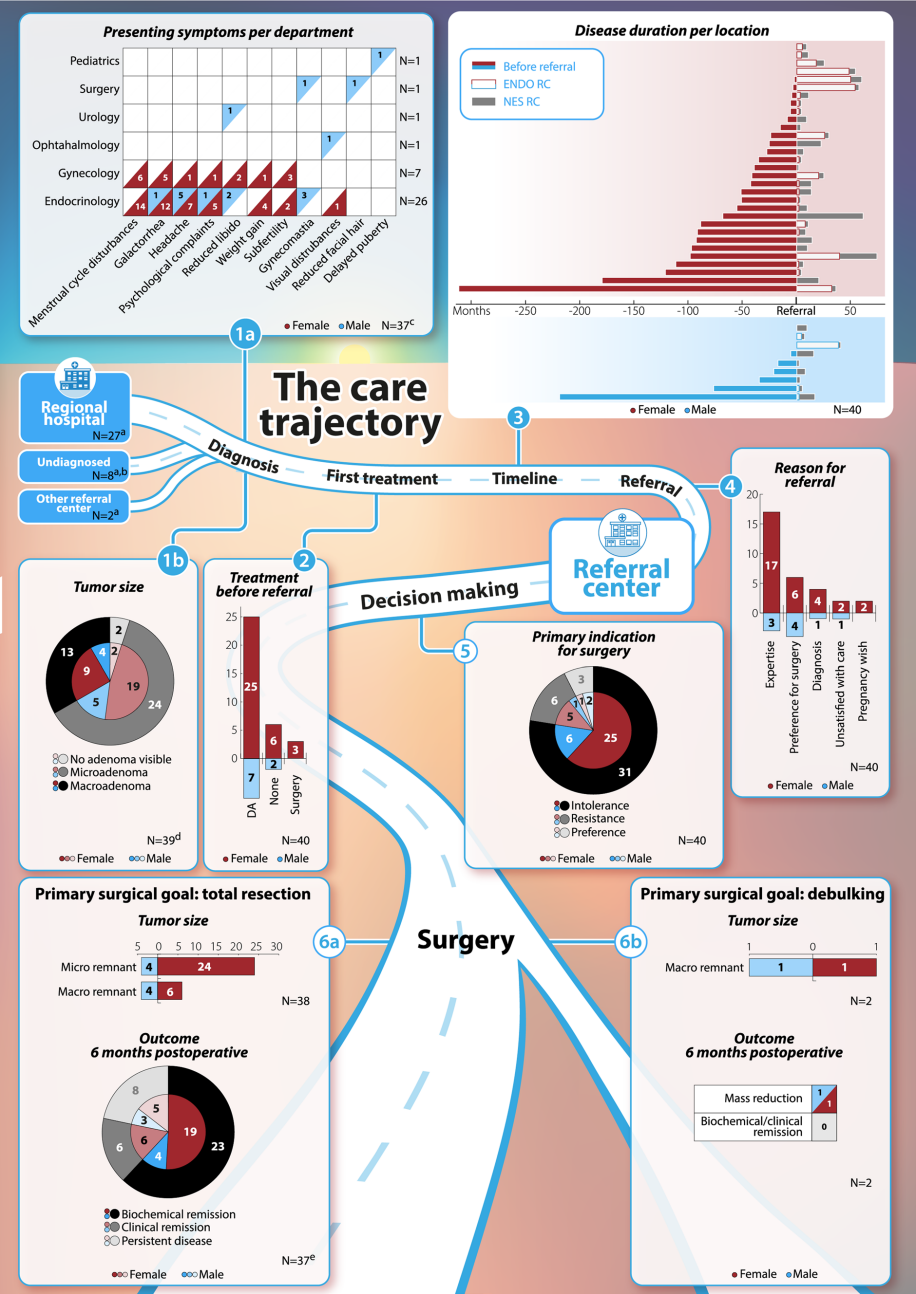
Summary of care trajectory for all patients

**Table 2:**
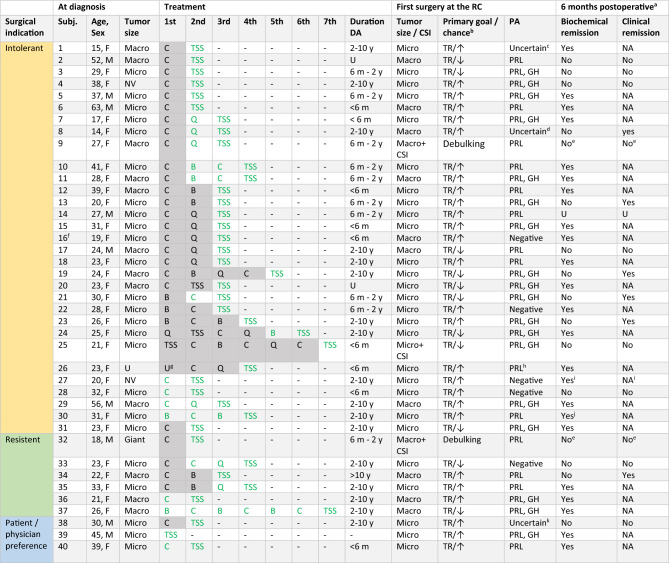
Demographics and treatment details per patient

Overview of demographical data, tumor characteristics, treatment details and outcomes per patient. The treatment trajectory till the first surgery at the RC was depicted. Patients may have undergone additional treatment after the first surgery at the RC. Compression of the optic chiasm was present in none of the patients at time of surgery. No permanent complications occurred. Biochemical remission was defined as normalization of prolactin. Clinical remission was defined as restoration of gonadal axis and resolution of symptoms, i.e. no indication for further treatment. *B* bromocriptine, *C* cabergoline, *CSI* cavernous sinus invasion, *F* female, *GH* growth hormone, *M* male, *Macro* macroadenoma, *Micro* microadenoma, *NV* not visible, *PA* histopathology, *PRL* prolactin, *Q* quinagolide, *RC* referral center, *subj.* subject, *TR* total resection, *TSS* transsphenoidal surgery, *U* unknown, ↑ optimal surgical chance for total resection, ↓ suboptimal surgical chance for total resection

Treatment undergone before referral to the RC

Treatment undergone at the RC

^a^6 months after the first surgery performed at the referral center

^b^Chance of achieving total resection for the patients in whom total resection was the primary surgical goal

^c^Hemorrhage and tissue that could be preexisting pituitary or adenoma with positive staining for ACTH, growth hormone and to a lesser extent prolactin

^d^No certain adenoma, small area with increased expression of prolactin and growth hormone

^e^Remission not expected as the goal of surgery was debulking

^f^Patient is a BAP1 gene mutation carrier

^g^Medication started in Poland, unknown which agent

^h^Dubious expression of growth hormone

^i^Remission status was measured 11 months postoperative

^j^Remission status measured 2 months postoperative, as the patient was lost to follow-up from this point on

^k^Uncertain adenoma, possible apoplexy

