# Supplementary material for: Care trajectories of surgically treated patients with a prolactinoma: why did they opt for surgery?
Source: Pituitary. 2023 Sep 10;26(5):611–21. doi: 10.1007/s11102-023-01346-z (PMC10539430; doi:10.1007/s11102-023-01346-z)
Supplement: Supplementary file 1 — Supplementary file1 (PDF 264 kb) [file 11102_2023_1346_MOESM1_ESM.pdf]

# Care trajectories of surgically treated patients with a prolactinoma: why did they opt for surgery?

## Supplements

Victoria R. van Trigt[1*], Ingrid M. Zandbergen[1*], Iris C.M. Pelsma[1], Leontine E.H. Bakker[1], Marco J.T. Verstegen[2], Wouter R. van Furth[2], Nienke R. Biermasz[1]

### Author affiliations:

1. Department of Medicine, Division of Endocrinology, and Center for Endocrine Tumors Leiden, Leiden University Medical Center, Leiden, the Netherlands
2. Department of Neurosurgery, Leiden University Medical Center, University Neurosurgical Center Holland, Leiden, The Netherlands.

   * Shared first authors

### ORCID:

V.R. van Trigt: 0000-0002-5658-0679
I.M. Zandbergen: 0000-0003-1077-8273
I.C.M. Pelsma: 0000-0003-4956-4037
M.J.T. Verstegen: 0000-0001-9739-7515
W.R. van Furth: 0000-0001-5208-921X
N.R. Biermasz: 0000-0001-5817-3594

### Corresponding author:

Name: V.R. van Trigt
Email: V.R.van_trigt@lumc.nl

## Supplementary Table 1 – Patient characteristics at first presentation to the RC

| | All patients, N=40 | Females, N=31 | Males, N=9 |
|---|---|---|---|
| **Disease parameters** | | | |
| Disease duration (years) | 2 (0-26) | 2 (0-26) | 1 (0-19) |
| Prolactin level (ULN) | 4.7 (0.8-100.0) | 3.7 (0.8-26.0) | 9.0 (1.3-100.0) |
| **Reason for referral** | | | |
| Expertise | 20 (50.0%) | 17 (54.8%) | 3 (33.3%) |
| Preference for surgery | 10 (25.0%) | 6 (19.4%) | 4 (44.4%) |
| Diagnosis | 5 (12.5%) | 4 (12.9%) | 1 (11.1%) |
| Unsatisfied with care | 3 (7.5%) | 2 (6.5%) | 1 (11.1%) |
| Pregnancy wish | 2 (5.0%) | 2 (6.5%) | 0 (0.0%) |
| **Treatment**[a] | | | |
| None | 24 (60.0%) | 21 (67.7%) | 3 (33.3%) |
| Cabergoline | 12 (30.0%) | 7 (22.6%) | 5 (55.6%) |
| Quinagolide | 2 (5.0%) | 1 (3.2%) | 1 (11.1%) |
| Bromocriptine | 2 (5.0%) | 2 (6.5%) | 0 (0.0%) |

Patient characteristics at the time of first presentation to the referral center for all patients and females and males separately. Values are presented as median (range) or number (percentage). *DA* dopamine agonist, *RC* referral center, *ULN* upper limit of normal.

[a] Active treatment at time of referral to the referral center

## Supplementary Table 2 – Patient characteristics at time of surgical treatment at the RC

| | | All patients N=40 | Females N=31 | Males N=9 |
|---|---|---|---|---|
| **Patient and tumor characteristics** | | | | |
| Age (years) | | 31.5 (18-65) | 31 (18-54) | 44 (18-65) |
| Disease duration (years) | | 4 (0-27) | 4 (0-27) | 2 (0-20) |
| Time between first consult at our RC and choice for surgery (days) | | 186 (23-2162) | 225 (27-2162) | 154 (23-1244) |
| Serum prolactin (ULN) | | 4.5 (0.2-81.4)[a] | 3.9 (0.2-44.0)[a] | 12 (1.3-81.4) |
| Suppression of FSH/LH | *Certain* | 17 (42.5%) | 9 (29.0%) | 8 (88.9%) |
| | *Possible* | 0 (0.0%) | 0 (0.0%) | 0 (0.0%) |
| Pituitary deficiency[b] | *Certain* | 6 (15.0%) | 4 (12.9%) | 2 (22.2%) |
| | *Possible* | 1 (2.5%) | 1 (3.2%) | 0 (0.0%) |
| ACTH | *Certain* | 1 (2.5%) | 0 (0.0%) | 1 (11.1%) |
| | *Possible* | 2 (5.0%) | 2 (6.5%) | 0 (0.0%) |
| GH | *Certain* | 0 (0.0%) | 0 (0.0%) | 0 (0.0%) |
| | *Possible* | 0 (0.0%) | 0 (0.0%) | 0 (0.0%) |
| TSH | *Certain* | 4 (10.0%) | 3 (9.7%) | 1 (11.1%) |
| | *Possible* | 1 (2.5%) | 1 (3.2%) | 0 (0.0%) |
| ADH | *Certain* | 1 (2.5%) | 0 (0.0%) | 1 (11.1%) |
| | *possible* | 0 (0.0%) | 0 (0.0%) | 0 (0.0%) |
| **MRI findings before surgery** | | | | |
| Tumor size | *Microadenoma* | 28 (70.0%) | 24 (77.4%) | 4 (44.4%) |
| | *macroadenoma* | 12 (30.0%) | 7 (22.6%) | 5 (55.6%) |
| | *Giant adenoma* | 0 (0.0%) | 0 (0.0%) | 0 (0.0%) |
| CSI | *KNOSP 3* | 2 (5.0%) | 2 (6.5%) | 0 (0.0%) |
| | *KNOSP 4* | 1 (2.5%) | 0 (0.0%) | 1 (11.1%) |
| Optic chiasma compression | | 0 (0.0%) | 0 (0.0%) | 0 (0.0%) |
| **Primary indication for surgery** | | | | |
| Intolerance | | 31 (77.5%) | 25 (80.6%) | 6 (66.7%) |
| Resistance | | 6 (15.0%) | 5 (16.1%) | 1 (11.1%) |
| Patient/physician preference | | 3 (7.5%) | 1 (3.2%) | 2 (22.2%) |

Details about transsphenoidal surgery for all patients and for females and males separately. Values are presented as median (range) or number (percentage). *GH* growth hormone, *CSI* cavernous sinus invasion, *RC* referral center.

[a] Nine patients were on dopamine agonist treatment, among which all patients with prolactin levels <1.0xULN.

[a] Deficiency of at least one pituitary hormone, excluding gonadotropic suppression

## Supplementary Table 3 – Details about surgical removal at the RC and outcomes

| Timepoint | | All patients | Females | Males |
|---|---|---|---|---|
| **Surgical details** | | **N=40** | **N=31** | **N=9** |
| Primary surgical goal | *Debulking* | 2 (5.0%) | 1 (3.2%) | 1 (11.1%) |
| | *Total resection* | 38 (95.0%) | 30 (96.8%) | 8 (88.9%) |
| Histopathology | *Confirmative* | 32 (80.0%) | 24 (77.4%) | 8 (88.9%) |
| | *Uncertain* | 4 (10.0%) | 3 (9.7%) | 1 (11.1%) |
| | *Negative* | 4 (10.0%) | 4 (12.9%) | 0 (0.0%) |
| **Outcomes 6 months postoperative** | | **N=39**[a] | **N=31** | **N=8**[a] |
| Biochemical remission | | 23 (55.0%)[b] | 19 (61.3%)[b] | 4 (50.0%) |
| Clinical remission | | 6 (10.2%)[b] | 6 (12.9%)[b] | 0 (0.0%) |
| **Long-term follow-up** | | **N=40** | **N=31** | **N=9** |
| Median duration of postoperative follow-up (months) | | 43 (2-71) | 44 (2-71) | 41 (13-59 |
| Additional treatment | *All types* | 13 (30.0%) | 9 (29.0%) | 4 (44.4%) |
| | *Reoperation onl*[c] | 3 (7.5%) | 1 (3.2%) | 2 (22.2%) |
| | *Medication only* | 4 (10.0%) | 3 (9.7%) | 1 (11.1%) |
| | *Reoperation and medication*[c] | 5 (12.5%) | 4 (12.9%) | 1 (11.1%) |
| Biochemical remission | *All* | 26 (65.0%) | 21 (67.7%) | 5 (55.6%) |
| | *Biochemically controlled on medication* | 3 (7.5%) | 3 (9.7%) | 0 (0.0%) |
| Clinical remission | | 4 (77.5%) | 4 (12.9%) | 0 (0.0%) |

Details about surgical removal of the prolactinoma at the referral center, short and long term outcomes of surgical treatment for all patients and for females and males separately. No permanent complications occurred. Values are presented as median (range) or number (percentage). *Biochemical remission* was defined as normalization of prolactin , *clinical remission* restoration of gonadal axis and resolution of symptoms i.e. no indication for further treatment. *RC* referral center.

[a] Missing data of one patients, as the patient refused blood tests for the period of 2.5 years. postoperatively, due to psychosocial problems. After 2.5 years prolactin was 1.6xULN.

[b] Remission status was measured 2 months postoperatively in one patient, who was lost to follow-up from this point on

[c] Of which one patient underwent two reoperations
